# Urotropin in Meningeal Infections, Especially in Acute Anterior Poliomyelitis

**Published:** 1910-12

**Authors:** 


### Urotropin in Meningeal Infections, Especially in Acute Anterior Poliomyelitis

SINCE the discovery by Cushing and Crowe (*Johns Hopkins Medical Bulletin,* April, 1908, and April, 1909) that urotropin is excreted in the cerebrospinal fluid and there manifests antiseptic properties, the then suggested use of this drug as a prophylactic routine measure in meningeal infections has found more and more general advocacy. The increased frequency with which epidemics of acute anterior poliomyelitis have made their appearance in various parts of the country, has brought forward numerous papers dealing with the prophylaxis and treatment of this disease, in which the use of urotropin in the early stages is strongly recommended.

Dr. M. Allen Star, Professor of Neurology, Columbia University, College of Physicians and Surgeons, New York, before the joint meeting of the N. Y. Neurological Society and the Section on Pediatrics of the N. Y. Academy of Medicine, May 4, 1909, (*Journal of Nervous and Mental Disease,* November, 1909) ; Dr. L. R. De Buys before the Louisiana State Medical Society (*New Orleans Medical and Surgical Journal,* March, 1910 ; Dr. Robert Preble, Professor of Medicine, Northwestern Medical School, Chicago, (*Journal of the American Medical Association,* September 24, 1910) ; Dr. Charles R. Ball, Instructor in Nervous Diseases, University of Minnesota (*St. Paul Medical Journal,* December, 1909) ; Dr. Colin K. Russell, of the Pathological Department, Royal Victoria Hospital, Montreal, Canada (*Montreal Medical Journal,* December, 1909) ; Dr. Haldor Sneve, St. Paul, Minnesota (*Journal of the Minnesota State Medical Association,* January, 1910 ; Dr. G. Stirling Landon, Late House Physician, Royal Infirmary, Edinburgh, and Queen's Hospital for Children (*London Lancet,* April 16, 1910) ; and Dr. Frederick E. Batten, Physician to the Hospital for Sick Children, London, England (*London Lancet,* June 18, 1910), have all declared themselves in favor of the employment of urotropin as a prophylactic agent in the treatment of meningeal infections.

Dr. Arthur R. Cushny, Professor of Pharmacology, University of London, says in the fifth edition (1910) of his "Text-book of Pharmacology and Therapeutics, or the Action of Drugs in Health and Disease," Lea & Febiger, Philadelphia and New York, "Numerous compounds of urotropin have been introduced of late by rival manufacturers, but none of these has proved equal to the original drug."

